# Triple-Negative Transformation in Hormone Receptor–Positive/Human Epidermal Growth Factor Receptor 2–Negative Metastatic Breast Cancer: Tissue Versus Liquid Biopsy in Two Cases

**DOI:** 10.1200/PO-26-00060

**Published:** 2026-09-23

**Authors:** Shahla Abu Naib, Paul Ellis, Eisa Velicaria, Crispin Hiley, Neill Patani, Humaid Al-Shamsi, Xiwen Wang, Darshana Patil, Vineet Datta, Kefah Mokbel, Tim Crook

**Affiliations:** ^1^ Leaders in Oncology Care, London, United Kingdom; ^2^ London Breast Institute, London, United Kingdom; ^3^ Institute for Women's Health, University College London, London, United Kingdom; ^4^ Burjeel Cancer Institute, Burjeel Medical City, Abu Dhabi, UAE; ^5^ Kings College London, London, United Kingdom; ^6^ Datar Cancer Genetics Ltd, Nashik, India; ^7^ Cromwell Hospital, London, United Kingdom

## Introduction

Precision oncology incorporates molecular profiling to guide personalized systemic therapy in metastatic breast cancer. Circulating tumor DNA (ctDNA) analysis has become widely adopted as a noninvasive means of detecting actionable genomic alterations and monitoring clonal evolution.^1^ Several clinical studies support its use to identify activating *ESR*1 mutations that confer resistance to aromatase inhibitors and may inform use of selective estrogen receptor (ER) degraders and PI3K or AKT inhibitors in hormone receptor–positive (HR+)/human epidermal growth factor receptor 2–negative (HER2−) disease.^2-4^ Clinical practice guidelines now endorse ctDNA testing when endocrine resistance is suspected.^5,6^

ctDNA has important limitations, however: shedding varies with tumor burden and metastatic site, most platforms cannot determine receptor expression, Ki-67 or PD-L1 status,^7,8^ and ctDNA represents a pooled signal that may disproportionately reflect minor subclones rather than the dominant clone driving progression.^8^

Recently developed methylation-based liquid biopsy approaches show promise for inferring tumor phenotype directly from blood. The Breast Circulating Methylation Assay, presented at the San Antonio Breast Cancer Symposium 2025, reportedly demonstrated approximately 90% accuracy in identifying receptor status switches, including transformation from ER-positive to triple-negative breast cancer.^9^ While promising, such approaches are likely subject to similar biological constraints as mutation-based ctDNA assays, including tumor heterogeneity and variable DNA shedding.

By contrast, image-guided tissue biopsy provides spatially resolved, lesion-specific information and remains the gold standard for receptor phenotyping and comprehensive genomic profiling although its implications for ctDNA-guided treatment selection are unclear.

We describe two patients with initially HR+/HER2- early breast cancer who developed metastatic relapse with triple-negative transformation. In both cases, sole reliance on ctDNA would have resulted in inappropriate systemic therapy selection. This highlights the need to combine tissue and liquid biopsy in metastatic breast cancer.

## Materials and Methods

### Study Design and Case Selection

This retrospective case series included two patients with HR+/HER2- early breast cancer and metastatic relapse, selected for (1) rapid progression on endocrine therapy, (2) serial ctDNA profiling, and (3) image-guided tissue biopsy at progression. Data were extracted from electronic medical records.

### Ethical Considerations

Both patients provided written informed consent for the use of anonymized clinical and molecular data for research and publication. This retrospective case series was conducted in accordance with the Declaration of Helsinki. As a retrospective case study, the Institutional Review Board determined that formal ethical committee approval was not required.

### Imaging Assessment

Serial fluorodeoxyglucose (FDG) positron emission tomography-computed tomography (CT), CT, and magnetic resonance imaging were performed. Progression was defined per RECIST v1.1 or metabolic criteria for bone-dominant disease.

### Tissue Biopsy and Histopathology

Image-guided core biopsies underwent immunohistochemistry for ER, progesterone receptor (PgR), HER2, Ki-67, and PD-L1 (22C3/SP142) and comprehensive genomic profiling (CellDx platform, Datar Cancer Genetics) per standard clinical protocols.

### Liquid Biopsy Analysis

#### Tumor-Agnostic ctDNA Profiling.

Plasma-based next-generation sequencing (NGS) was performed using Guardant360 and Exacta (single-nucleotide variants, indels, copy number alterations and fusions). Mutant allele frequency (MAF) was reported per variant; paired germline sequencing excluded CHIP artifacts.

#### Tumor-Informed MRD Testing.

Tumor-informed minimal residual disease (MRD) was quantified by multiplex digital droplet PCR using patient-specific assays (eg, *TP53* p.R248Q, *GATA3* c.991dup), reported as mutant copies/mL.

### Cross-Sample Concordance of Tumor-Associated Alterations

Tissue and plasma assay concordance was evaluated across shared panel regions; discordance was attributed to biological rather than technical differences.

### Sequencing Quality Control and Decalcification-Related Artifact Mitigation

All sequencing data underwent stringent bioinformatic filtering (strand bias, base quality, deamination artifacts) with custom formalin-fixed paraffin-embedded/decalcification filters. Predefined thresholds included coverage >1,500×, on-target uniformity >90%, median absolute pairwise difference <0.5, and an artifact-aware tumor mutational burden (TMB) framework.

### Informed Consent Statement

Written informed consent was obtained from both patients for use of anonymized clinical, imaging, and molecular data for publication.

## Results

### Case 1

A 44-year-old premenopausal woman was diagnosed with grade III ER+/progesterone receptor (PR+)/HER2- T2N1 invasive ductal carcinoma (right breast) in Month 1. Germline analysis was negative. She received mastectomy, axillary dissection and adjuvant chemotherapy (EC × 4/paclitaxel × 7), radiotherapy, and tamoxifen from Month 12.

In Month 28, rising Ca15-3 led to staging; sacral biopsy (Month 31) confirmed *GATA3*-positive metastatic carcinoma (ER 8, PR 0, HER2 0; Ki-67 30%).

Prebiopsy liquid biopsy results are shown in Table 1 (Section A).

**TABLE 1.** Molecular Profiling Data for Case 1

Gene	Variant	MAF (%)
Section A: Tumor-agnostic liquid biopsy—Case 1 (Month 30)		
*NOTCH1*	I2089M	0.22
*RB1*	R455*	5.82
*TP53*	R248Q	21.38
*EGFR*	Amplification	2.85
Section B: Tumor-agnostic liquid biopsy—Case 1 (Month 39)		
*TP53*	R248Q	19.77
*ESR1*	Y537N	6.14
*PIK3CA*	H1047R	5.36
*PIK3CA*	E545K	0.75
Section C: Tissue genomic profiling—Case 1, metastatic bone biopsy (Month 39)		
*RB1*	c.1363C>T p.R455*	26.9
*TP53*	c.743G>A p.R248Q	48.98
*NF1*	c.5731_5738del p.I1911Dfs*6	23.79
*ATR*	c.2057_2058delAC p.N686Kfs*9	27.09
*ATR*	c.5739-14_5739-6delinsT	41.73
*MGA*	Amplification	6 copies
*BCL2L12*	Amplification	12 copies
*POLD1*	Amplification	6 copies
*PPP2R1A*	Amplification	12 copies
*AURKC*	Amplification	11 copies
Section D: Tissue genomic profiling—Case 1, metastatic bone biopsy (Month 50)		
*NF1*	p.I1911Dfs*6	30.26
*RB1*	p.R455*	38.89
*TP53*	p.R248Q	57.55
*ATR*	p.N686Kfs*9	26.15
*VHL*	p.E17*	17.25
*SDHB*	p.R115Q	8.78
*JAK1*	p.S404F	19.49
*RUNX1T1*	p.S60C	16.16
*CYP2C9*	p.R97K	28.79
*ATM*	p.D2987H	8.15
*TBX3*	p.S244C	45.9
*MGA*	p.R2466G	5.21
*CREBBP*	p.G284A	5.05
*STAT3*	p.E407K	15.14
*BRCA1*	c.213-30G>A	8.94
*PIK3R2*	p.E24Q	8.33
*SMARCB1*	p.Q323H	6.7
*CARD11*	p.Q460*	13.97
*KMT2C*	p.H4506Q	32.51
*BRCA2*	p.D762N	14.87
*FANCM*	p.R1456C	65.15
*ATR*	c.5739-14_5739-6delinsT	31
*EGFR*	Amplification	6 copies
*PPP2R1A*	Amplification	7 copies
*BCL2L12*	Amplification	7 copies
*AURKC*	Copy number gain	4 copies
*MGA*	Copy number gain	5 copies

NOTE. Month 1 denotes the date of initial diagnosis for Case 1; all other time points in Table 1 and Figures 1A and 1B are expressed in months elapsed since diagnosis. Month numbering is defined independently for each case (see also Table 2 for Case 2), and month numbers do not correspond between cases.

Abbreviation: MAF, mutant allele frequency.

First-line systemic therapy with goserelin, letrozole, ribociclib, and denosumab was commenced in Month 31 following a course of palliative radiotherapy to a symptomatic skull vault metastasis. There was initial response to first-line systemic therapy, but in Month 39, the serum Ca15-3 was rising and FDG PET-CT showed extensive bone-predominant progressive metastatic disease.

Repeat liquid biopsy was performed (Table 1, Section B), which redemonstrated the presence of *TP53* R248Q, but new variants in *ESR1* (Y537N) and two variants in *PIK3CA* (H1047R and E545K) were now present, consistent with the development of resistance to aromatase inhibition.

Tissue biopsy (Month 39) demonstrated *GATA3*-positive carcinoma with triple-negative phenotype (ER 0, PgR 0, HER2 0; Ki-67 30%; PD-L1 negative; tumor cellularity 25%). Genomic profiling is summarized in Table 1 (Section C).

*TP53* R248Q and *RB1* R455* were confirmed on tissue, but *ESR1* and *PIK3CA* variants from the concurrent liquid biopsy were absent. Instead, novel *ATR* and *NF1* alterations were identified. High truncal MAFs (TP53 48.98%, RB1 26.9%) confirmed adequate tumor representation.

In view of the transformation in receptor expression phenotype and PD-L1–negative disease, systemic therapy was changed to nab-paclitaxel plus bevacizumab, with stereotactic ablative radiotherapy (SABR) to symptomatic spinal metastasis.

PET-CT after two cycles confirmed a near-complete metabolic response.

Consistent with response, tumor-informed ctDNA analysis demonstrated a reduction in *TP53* c.743G>A from 249 copies/mL at Month 39 to 2.3 copies/mL at Month 43 and tumor-agnostic NGS showed a fall in MAF from 19.77% to 0% (Figs 1A and 1B).

**FIG 1.**
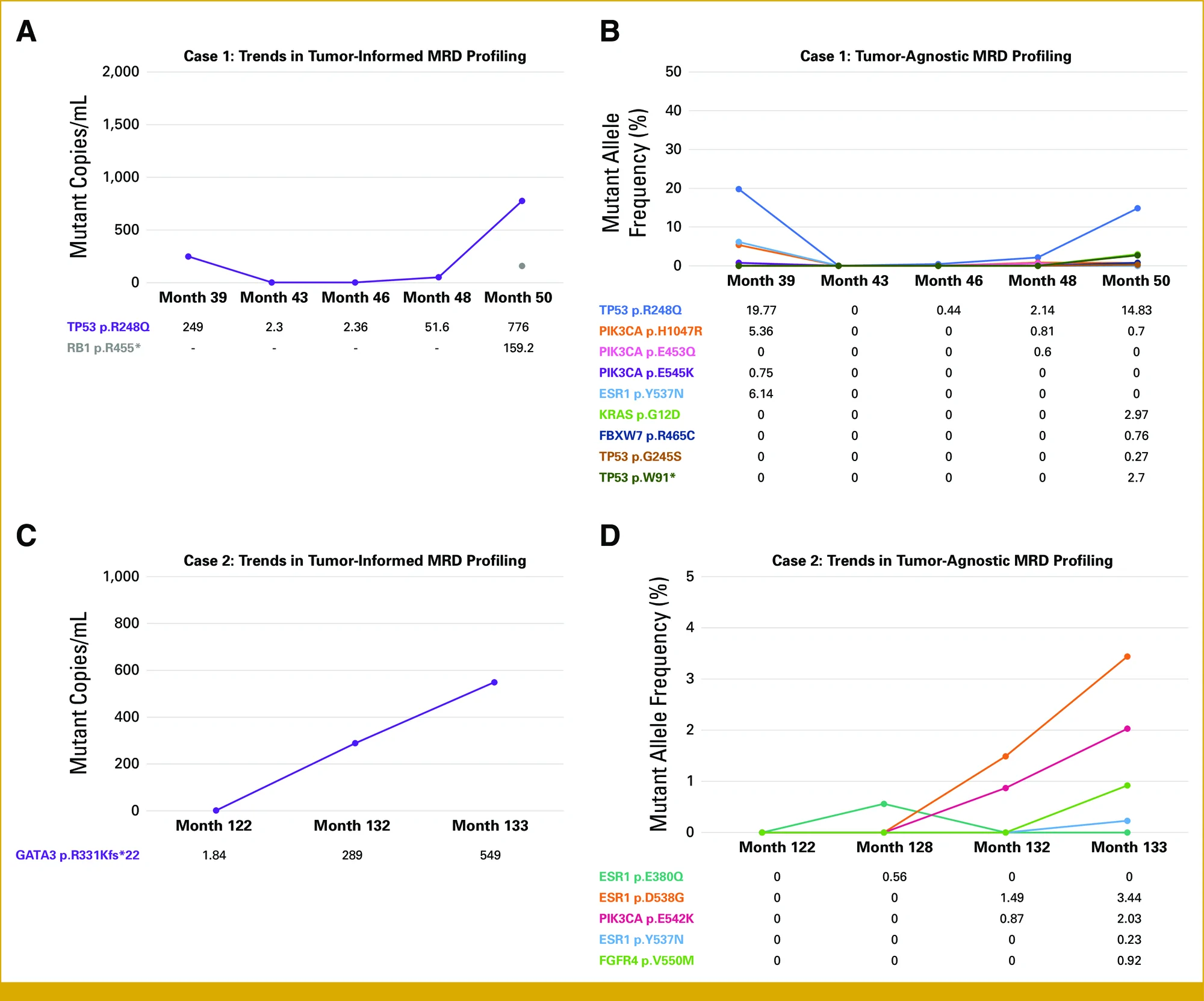
Longitudinal ctDNA dynamics: (A and B) Case 1 and (C and D) Case 2. Case 1: (A) Tumor-informed MRD profiling showing temporal changes in *TP53 p.*R248Q and *RB1 p.*R455* mutant copies/mL across serial samples. (B) Tumor-agnostic MRD profiling showing mutant allele frequencies for *TP53*, *PIK3CA*, *ESR1*, and other detected variants, illustrating emergence of endocrine-resistant subclones. *NOTCH1* and *RB1* variants (Month 30) were not assessed in all subsequent assays and are therefore not directly comparable. Dashes (–) indicate variants not evaluated at specific time points. Month 1 for each case denotes the date of initial diagnosis; Case 1 and Case 2 are staged independently, and month numbers are not on a shared calendar timeline (see Tables 1 and 2). Case 2: (C) Tumor-informed MRD profiling showing rising *GATA3* c.991dup mutant copies/mL corresponding to increasing disease burden. (D) Tumor-agnostic MRD profiling showing sequential emergence of *ESR1*, *PIK3CA*, and *FGFR4* alterations consistent with developing aromatase inhibitor resistance. MRD, minimal residual disease.

After disease recurrence at Month 50 (multiple sites), SABR was delivered to high-risk lesions and CT-guided right iliac crest biopsy was performed (tumor cellularity 35%).

Histologic evaluation demonstrated *GATA3*-positive carcinoma with re-expression of ER, with a phenotype ER 8, PgR 0, and HER2 0 with Ki-67 40%. PD-L1 analysis using both 22C3 and SP142 antibodies remained negative. Despite ER re-expression, comprehensive tissue genomic profiling continued to demonstrate pathogenic alterations in *TP53*, *RB1*, *ATR*, and *NF1*, with no detectable *ESR1* or *PIK3CA* mutations, indicating phenotypic plasticity.

Concurrent liquid biopsy showed re-emergence of *PIK3CA* p.H1047R, consistent with a spatially distinct HR-positive subclone not represented in the biopsied lesion.

Treatment was switched to fulvestrant plus capivasertib with ongoing bone support.

Repeat tissue genomics revealed increased complexity: TMB rose from 4 to 33 mutations/Mb; *BRCA1/2* variants of uncertain significance were identified, reflecting genomic instability under therapeutic pressure. Full findings are provided in Table 1 (Section D).

### Case 2

A 21-year-old premenopausal woman was diagnosed with T1cN1 grade II ER+/PgR+/HER2- invasive ductal carcinoma (left breast) at Month one and received adjuvant EC × 4/paclitaxel × 12, radiotherapy, and goserelin/tamoxifen (tamoxifen until Month 93).

Bone-predominant metastatic recurrence was confirmed on T8 biopsy at Month 102 (*GATA3*+, ER 8, PgR 8, HER2 0).

First-line systemic therapy with goserelin, letrozole, and ribociclib (with denosumab) was initiated at Month 104, and complete metabolic remission was achieved.

Serial liquid biopsy identified *ESR1* E380Q (Month 128) and then *ESR1* D538G, *PIK3CA* E542K, and *FGFR*1 amplification (Month 132), consistent with aromatase inhibitor resistance. A complete chronology is in Table 2.

**TABLE 2.** Sequential ctDNA Alterations for Case 2 (Months 122-134)

Date	Gene	Variant	MAF (%)
Month 122	—	None detected	0
Month 128	*ESR1*	E380Q	0.56
Month 132	*ESR1*	D538G	1.49
Month 132	*PIK3CA*	E542K	0.87
Month 132	*FGFR1*	Amplification	Detected
Month 133	*ESR1*	D538G	3.44
Month 133	*ESR1*	Y537N	0.23
Month 133	*PIK3CA*	E542K	2.03
Month 133	*FGFR4*	V550M	0.92
Month 133	*FGFR1*	Amplification	Detected

NOTE. Month 1 denotes the date of initial diagnosis for Case 2; all other time points in Table 2 and Figures 1C and 1D are expressed in months elapsed since diagnosis. Month numbering is defined independently for Case 1 and Case 2 (see Table 1), and month numbers do not correspond between cases.

Abbreviations: ctDNA, circulating tumor DNA; MAF, mutant allele frequency.

An updated FDG PET-CT at Month 133 showed new liver lesions and multiple new bone metastases. Based on the presence of *ESR1* D538G and *PIK3CA* E542K, next-line systemic therapy options included fulvestrant with alpelisib, fulvestrant with capivasertib, or fulvestrant with continued CDK4/6 inhibition.

The patient was switched to fulvestrant and ribociclib, but serum Ca15-3 continued to rise, with imaging confirming further progression.

A follow-up PET-CT at Month 134 confirmed progressive disease in both bone and liver. Liver biopsy demonstrated high-grade triple-negative carcinoma (ER 0, PgR 0, HER2 0; Ki-67 80%; PD-L1–negative), establishing clonal evolution from the original HR-positive phenotype.

Tumor-informed MRD demonstrated rising *GATA3* c.991dup mutant copies/mL (1.84 → 289 → 549), corresponding closely with the appearance of endocrine-resistant subclones on ctDNA and preceding radiographic progression (Figs 1C and 1D).

## Discussion

We present two patients with HR+/HER2- metastatic breast cancer who progressed unexpectedly on first-line endocrine-based therapy, prompting tissue rebiopsy that fundamentally altered management.

In Case 1, ctDNA at progression identified *ESR1* (Y537N) and *PIK3CA* (H1047R, E545K) mutations consistent with endocrine resistance, suggesting elacestrant-, capivasertib-, or alpelisib-based therapy.^3,10,11^ However, concurrent tissue biopsy revealed triple-negative transformation (ER 0/PgR 0/HER2 0) and novel *ATR* and *NF1* mutations, confirming a biologically distinct dominant clone not appreciable from ctDNA alone.

In Case 2, after approximately 2 years of disease control, ctDNA revealed emerging *ESR1*, *PIK3CA*, and *FGFR1* alterations; liver biopsy at progression confirmed triple-negative transformation that could not be inferred from ctDNA alone.

In both cases, early endocrine failure reflects clonal replacement by triple-negative clones. Truncal mutations (*TP53* R248Q/*RB1* R455* in Case 1; *GATA3* c.991dup in Case 2) support clonal selection; *RB1* R455* predicts CDK4/6 inhibitor resistance.^12^ *ESR1*/*PIK3CA* mutations in ctDNA likely represent persisting aromatase inhibitor–resistant subclones.^13,14^ Crucially, sole reliance on ctDNA risks misrepresenting dominant disease biology and selecting inappropriate therapy.

The later evolution of Case 1 illustrates phenotypic plasticity: despite ER re-expression after chemotherapy, tissue genomics showed persistent *TP53*, *RB1*, *ATR*, and *NF1* alterations (no *ESR1*/*PIK3CA*), whereas ctDNA again identified *PIK3CA* variants. High truncal MAFs (*TP53* 57.55%, *RB1* 38.89%) exclude low tumor cellularity; this phenotype-genotype discordance confirms that ctDNA alone cannot define the genomic drivers of dominant disease.

Optimal therapy selection requires concurrent tissue and ctDNA profiling; image-guided biopsy provides spatially resolved genomic information that ctDNA cannot reliably supply.^13,14^

These findings have practice-changing implications: current guidelines prefer plasma ctDNA for *ESR*1 testing at progression,^5,6^ the NHS Genomic Medicine Service is implementing this,^15^ and elacestrant's license (KORSERDU) requires plasma-based *ESR1* identification only.^16^

Tissue biopsy of new or progressive lesions remains essential and should be integrated into routine assessment at treatment failure, particularly when clinical behavior is discordant with ctDNA findings.

This retrospective case series (N = 2) is limited by sample size, selection bias, and single-site sampling. Whether *ESR1* and *PIK3CA* mutations in ctDNA arose from minor subclones, spatially segregated lesions, or transitional phenotypes could not be determined. Nevertheless, these cases provide clear biological and clinical evidence that ctDNA may not reliably reflect the dominant disease-driving clone.

## Data Availability

All data generated or analyzed during this study are included within the article. Additional clinical or molecular details may be made available from the corresponding author upon reasonable request.
